# Backbone Cyclization and Dimerization of LL-37-Derived Peptides Enhance Antimicrobial Activity and Proteolytic Stability

**DOI:** 10.3389/fmicb.2020.00168

**Published:** 2020-02-21

**Authors:** Sunithi Gunasekera, Taj Muhammad, Adam A. Strömstedt, K. Johan Rosengren, Ulf Göransson

**Affiliations:** ^1^Pharmacognosy, Department of Medicinal Chemistry, Biomedical Centre, Uppsala University, Uppsala, Sweden; ^2^School of Biomedical Sciences, The University of Queensland, Brisbane, QLD, Australia

**Keywords:** peptide cyclization, peptide dimerization, antimicrobial peptide, host defense, KR-12, LL-37

## Abstract

Can antimicrobial activity and peptide stability of alpha-helical peptides be increased by making them into dimers and macrocycles? Here, we explore that concept by using KR-12 as the starting point for peptide engineering. KR-12 has previously been determined as the minimalized antimicrobial fragment of the human host defense peptide LL-37. Backbone-cyclized KR-12 dimers, tethered by linkers of two to four amino acid residues, were synthesized and their antimicrobial activity, proteolytic stability and structures characterized. A modified KR-12 sequence, with substitutions at previously identified key residues, were also included in the screening panel. The backbone cyclized KR-12 dimers showed improved antimicrobial activity and increased stability compared to monomeric KR-12. The most active cyclic dimer displayed 16-fold higher antibacterial activity compared to KR-12 against *Pseudomonas aeruginosa* and *Staphylococcus aureus*, and 8-fold increased fungicidal activity against *Candida albicans*. It also showed increased hemolytic and cytotoxic activity. Enhanced antimicrobial activity coincided with increased membrane permeabilization of liposomes with one distinct discrepancy: monomeric KR-12 was much less disruptive of liposomes with bacterial lipid composition compared to liposomes from fungal lipid extract. Circular dichroism showed that the four-residue linked most active cyclic dimer had 65% helical content when bound to lyso-phosphatidylglycerol micelles, indicating that the helical propensity of the parent peptide is maintained in the new macrocyclic form. In conclusion, the current work on KR-12 suggests that dimerization together with backbone cyclization is an effective strategy for improving both potency and stability of linear antimicrobial peptides.

## Introduction

**FIGURE 1:**
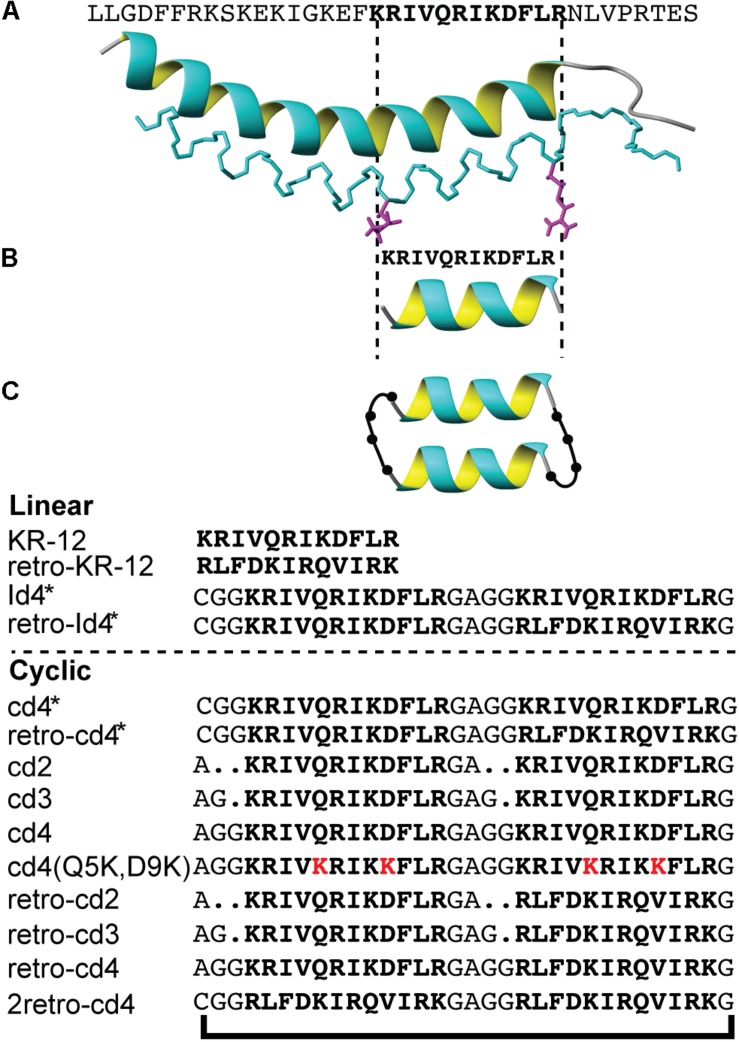
The design strategy of KR-12 cyclic analogs. **(A)** The three-dimensional structure of LL-37 (PDB code 2K60), **(B)** the minimum antibacterial region KR-12, **(C)** a schematic representation of the backbone cyclized KR-12 dimer. Sequences of all analoges are provided.

Antimicrobial peptides (AMPs) are gaining interest for antibiotic discovery and development, but despite natural functions as host defense peptides they come with inherent challenges in stability and potency. Here, we explore the concept of making a short alpha-helical peptide into a dimer, which in turn is made into a macrocycle to address these challenges (Figure 1). Specifically, does dimerization increase activity and can we then improve stability and activity further by macrocyclization? The 12-residue long peptide epitope named KR-12 was subjected to peptide engineering to answer these questions.

KR-12 is the shortest antimicrobial epitope of the human host defense peptide LL-37. The parent peptide is the only human cathelicidin, and it displays broad-spectrum antimicrobial activity (Larrick et al., 1993; Sochacki et al., 2011; Barns and Weisshaar, 2013) and immunomodulatory properties (Scott et al., 2002; Burton and Steel, 2009; Vandamme et al., 2012). LL-37 is also reported to inhibit biofilms (Overhage et al., 2008; Mishra et al., 2016), induce chemotaxis of immune cells (Agerberth et al., 2000), and promote wound healing (Heilborn et al., 2003; Koczulla et al., 2003). These activities have led LL-37 into the clinic: topical treatment of venous leg ulcers with LL-37 displayed significant wound healing effect in Phase I/II trials (Grönberg et al., 2014); and a 24-mer derivate (OP-145) has been investigated in Phase II clinical trials for treatment of chronic bacterial middle-ear infection (Fjell et al., 2011). Furthermore, LL-37 is currently undergoing Phase I/II trials for its immunomodulatory role in melanoma progression (Dolkar et al., 2018)^1^. However, the direct use of this 37-amino acid long alpha-helical peptide is hampered by protease susceptibility, in particular for antimicrobial applications. Engineering of LL-37, or peptides derived thereof, into more biologically stable forms would further improve its potential for clinical use (Schmidtchen et al., 2002; Strömstedt et al., 2009a). In the current work, we describe the development of such an improved peptide based on residues 18-29 of LL-37; KR-12.

Different truncated peptides derived from LL-37 have been subjected to antimicrobial (Li et al., 2006), biofilm inhibition (Feng et al., 2013; Mishra et al., 2016; Wang et al., 2017), anti-HIV (Wang et al., 2008) and anti-cancer (Li et al., 2006) assays to reveal structure-activity relationships. The N-terminal fragment LL-12, i.e., residues 1–12 of LL-37, is inactive against bacteria and cancer cells, whereas the C-terminal (residues 17-29) retains the core antimicrobial and anticancer activities of LL-37 (Li et al., 2006; Ren et al., 2013). A glycine-appended FK-16 peptide, referred to as GF-17, is more active than full-length LL-37 against both planktonic and biofilm forms of *Staphylococcus aureus* (Wang et al., 2012). The cytotoxicity of the C-terminal domain of LL-37 triggered the search for other epitopes, resulting in the identification of KR-12 (Figure 1), which was reported to be as active as LL-37 (MIC 40 μM) against *Escherichia coli*, accompanied by low cytotoxicity (Wang, 2008).

In our hands, KR-12 displayed a MIC of 2.5 μM against *Escherichia coli* and 10 μM against other tested pathogens, i.e., *Pseudomonas aeruginosa*, *Staphylococcus aureus*, and *Candida albicans* (Gunasekera et al., 2018). In that work, Ala and Lys scans of KR-12 showed that replacement of hydrophobic and cationic residues with Ala were detrimental for antimicrobial potency. Substitutions with Lys increased activity as long as the resulting increase in cationic density did not disrupt the amphiphilic disposition of the helical structure. Key positions were identified as Gln5 and Asp9, which when replaced with Ala or Lys increased broad-spectrum activity up to 8-fold against *S. aureus*, *P. aeruginosa*, and *C. albicans* (Gunasekera et al., 2018). In line with previous work (Wang, 2008; Wang et al., 2008), KR-12 showed weak cytotoxic activity with only a 13% loss of human cell line viability at the maximum concentration of 80 μM. In comparison, LL-37 displayed distinct cytotoxicity in this assay, having an IC50 of 10 μM. Importantly, the beneficial mutations of Gln5 and Asp9 were well tolerated, and neither of the double substituted analogues Q5A,D9A and Q5K,D9K showed any substantial cytotoxicity within the concentration interval used (Gunasekera et al., 2018). All the above indicated that KR-12 was an attractive starting point for peptide engineering.

Although the antibacterial domain of LL-37 can be condensed to KR-12, its susceptibility to enzymatic degradation cannot be prevented as multiple enzyme cleavage sites are still harbored within the sequence. LL-37 has been shown to be rapidly degraded by culture supernatants of *Bacillus anthracis*, presumably due to the action of secreted proteases that provides pathogen resistance (Schmidtchen et al., 2002; Thwaite et al., 2006). Components of gingival crevicular fluid rapidly degrade LL-37, limiting its role as a potential therapeutic for periodontitis (McCrudden et al., 2013). Truncated peptides derived from LL-37 have been shown to lose their bactericidal effect upon overnight incubation with chymotrypsin (Wang et al., 2014). Terminal modifications and introduction of bulky side-chains (Trp) in the internal fragment EFK-17 (residues 16–33, including KR-12) improved protease resistance against some bacterial proteases (Strömstedt et al., 2009a). However, the stability gained through introduction of D-amino acids at the same key positions rendered the peptide without activity, emphasizing the importance of not hindering helical propensity. Other examples of strategies that has been tested in order to increase peptide stability for other AMPs include using all-D-amino acids (Wade et al., 1990), β-peptides and α-peptoids (Godballe et al., 2011) or by conjugating methoxyethylglycine chains to the terminals (Park et al., 2011).

Backbone cyclization has also emerged as a technique to prevent protease susceptibility; several examples are found where cyclization has improved peptide stability either via naturally existing cyclic peptide scaffolds or by ligating the N- and C- termini of linear unstable peptides (Gunasekera et al., 2008; Clark et al., 2010; Chan et al., 2011). Full length LL-37 itself has been head-to-tail cyclized and shown to have similar antibacterial and antifungal activity as linear LL-37 (Kamysz et al., 2012).

The aim of the present work was to explore the concept of backbone cyclization and dimerization as means to enhance peptide stability and activity, in the pursuit of potent and stable AMPs based on KR-12. At the same time, this approach addressed the question of how an increased local peptide concentration at the membrane, achieved by “pre-formed dimers” of KR-12 with covalent linkers of variable length, will affect membrane permeabilization. The permeabilization efficacy on microbial membranes by engineered KR-12 analogs, their structure-activity relationships, as well as their stability, were examined to demonstrate the potential of this strategy for AMP drug development.

## Results

### Design and Synthesis of Linear and Cyclic Dimers

**FIGURE 2:**
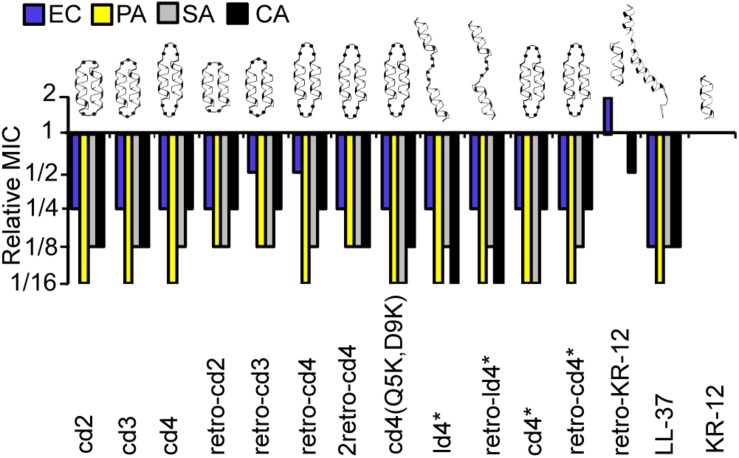
Relative MICs of the KR-12 dimers. The colors/patterns represent the organisms: *E. coli* (blue), *P. aeruginosa* (yellow) *S. aureus* (gray), *C. albicans* (black). In the naming of the dimers, either the prefix ‘c’ or ‘l’ were used to indicate that the dimers were cyclic or linear, respectively. Dimers were distinguished from monomers by ‘d’ and the number of residues in the linker was also included in the prefix. For example, peptide cd2 is a cyclic dimer of two KR-12 units connected one after the other with two-residue linkers between adjoining C- and N- termini of the counterpart monomers. Peptides cd3 and cd4 are similar to cd2 except that the size of the linker is three and four residues respectively. The term ‘retro’ was included in the name to highlight those analogs with two monomeric units adjoined in retro direction to each other. MIC values of KR-12 are *E. coli*: 0.312 μM; *P. aeruginosa*: 0.625 μM; *S. aureus*: 1.25 μM; *C. albicans* 1.25 μM. For cd4(Q5K,D9K), values are 0.625, 0.625, 0.625, and 1.25 μM, respectively. All Median MIC values can be found in Table 1.

A series of KR-12 derivatives were synthesized to investigate whether the antimicrobial potency and biological stability can be enhanced by dimerization and head-to-tail cyclization. Peptides were designed to determine the importance of linker length (two, three, or four residues) joining the monomers and how it affects the efficiency of peptide cyclization and activity. Moreover, the design strategy included tandem repeats (i.e., a dimer formed by two KR-12 sequences) and reverse tandem repeats (formed by KR-12 followed by retro-KR-12 or two retro-KR-12 sequences). The series of peptides and their nomenclature are detailed in Figure 2.

**TABLE 1:** Minimum inhibitory concentration (MIC) of peptides.

Peptide	MIC (μ M)			
	*E. coli*	*P. aeruginosa*	*S. aureus*	*C. albicans*
LL-37	0.312	0.625	1.25	1.25
KR-12	2.5	10	10	10
retro-KR-12	5	10	10	5
retro-cd4	1.25	0.625	1.25	2.5
retro-cd3	1.25	1.25	1.25	2.5
retro-cd2	0.625	1.25	1.25	2.5
cd4	0.625	0.625	1.25	2.5
cd3	0.625	0.625	1.25	1.25
cd2	0.625	0.625	1.25	1,25
cd4-(Q5K,D9K)	0.625	0.625	0.625	1.25
2retro-cd4	0.625	1.25	1.25	2.5
retro-ld4*	0.625	0.625	1.25	0.625
ld4*	0.625	0.625	1.25	0.625
retro-cd4*	0.625	0.625	0.625	2.5
cd4*	0.625	0.625	1.25	2.5

Peptides intended for cyclization were synthesized as linear chains including a C-terminal diaminobenzoic acid (Dbz) group and an N-terminal Cys following an established protocol (Gunasekera et al., 2013). The Dbz group was later converted into an *N*-benzymidazolinone (Nbz) group to facilitate peptide backbone cyclization by native chemical ligation (Dawson and Kent, 2000; Gunasekera et al., 2013). The Cys residue needed for that reaction was subsequently converted into Ala (Wan and Danishefsky, 2007). No Nbz peptide or hydrolysis product could be observed following incubation in the cyclization buffer, confirming that all dimers were cyclized efficiently irrespective of whether the dimer consisted of two KR-12 repeats, a KR-12 and a retro-KR-12, or two retro-KR-12. Furthermore, the length of the linker did not influence the efficiency of cyclization. MS analysis showed no evidence of intermolecular disulfide bond formation between dimers upon incubation in the cyclization buffer. Following desulfurization, no starting Cys containing peptide was observed, indicating that desulfurization was complete. The net charge, hydrophobicity, expected and observed masses and yields of cyclic KR-12 dimers are shown in Supplementary Tables 1, 2. Analytical HPLC chromatogram of final products are shown in Supplementary Figure 1, demonstrating purities of >95%.

### Dimerization of KR-12 Increases Antimicrobial Activity

The relative MICs of the analogs (ratio between the MICs of the analogs and MIC of KR-12) were compared to evaluate the influence on dimerization and cyclization on antimicrobial activity (Figure 2). A complete list of median MIC values is available in Table 1. Covalent linking of monomers resulted in substantial increase in antimicrobial activity compared to KR-12, irrespective of whether they were head-to-tail cyclized or linear and irrespective of cysteines to alanine conversions. Most dimers reached an activity level equivalent to, or surpassing, LL-37. The increase of antibacterial activity from dimerization was particularly high with regards to *P. aeruginosa*, typically reaching MIC at 16-fold lower concentration than the monomeric KR-12. Most analogs also displayed similar antibacterial activity as LL-37 against *S. aureus*, i.e., an 8-fold improvement in activity compared to KR-12.

Out of all the dimers, cd4(Q5K,D9K) stood out as the overall most active peptide, with 16-fold lower MICs against both *P. aeruginosa* and *S. aureus*, and 8-fold lower MIC against *C. albicans*. Notably, the activity of cd4(Q5K,D9K) against *S. aureus*, surpassed that of LL-37. Dimerization seems to have made least improvement to activity against *E. coli*: the maximum increase compared to KR-12 was 4-fold improvements and no dimer was as active as LL-37.

Reversing the sequence into a retro-KR-12 monomer had marginal effect on activity, a slight decrease in effect on *E. coli*, but an increase on *C. albicans*. However, the tendency of increased activity on *C. albicans* was much more pronounced when comparing the linear dimers ld4^∗^ and retro-ld4^∗^, the latter having 16-fold lower MIC. Overall, dimerization proved to be favorable for antifungal activity with 4 to 16-fold improvement to the activity as compared to KR-12.

Cyclization by itself does not appear to influence antibacterial activity. For example, the linear dimers and cyclic dimers both gave 8 to 16-fold improvement in activity against *S. aureus* and *P. aeruginosa.* For antifungal activity, linear dimers were 4-fold more active compared to their cyclized counterparts. No clear connection between activity and length of linkers or direction of the sequences could be identified.

### Bacterial and Fungal Liposome Permeabilization

**FIGURE 3:**
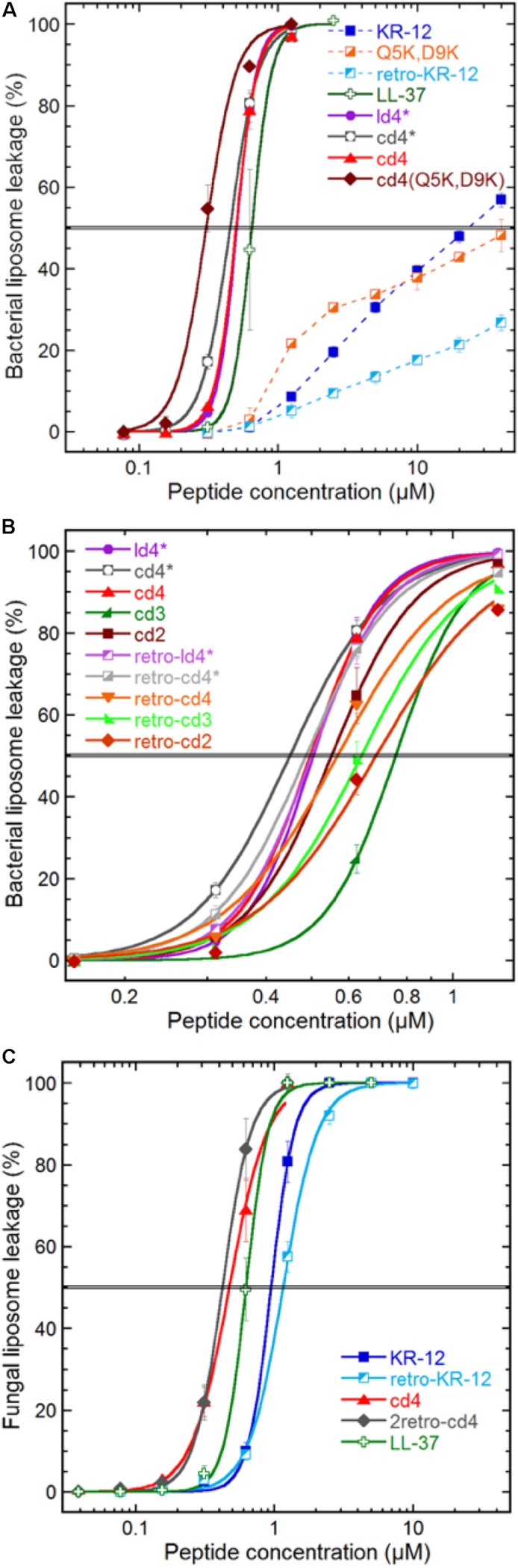
Peptide induced liposome leakage. Bacterial liposomes based on *E. coli* polar lipid extract **(A,B)**, and fungal liposomes of *S. cerevisiae* polar lipid extract and ergosterol (7:3) **(C)**, were monitored for fluorescent marker leakage over 45 min of incubation with peptides. **(A)** Compares membrane disrupting efficacy at the 45 min mark for the parent peptide, monomer, lysine substitutions and dimerization, **(B)** effects of linker length, monomer directionality and backbone cyclization, **(C)** effect on KR-12 directionality and dimerization on a fungal liposome system. The “^∗^” indicates that the cysteine in the ligation-linker has not been converted to an alanine, in order to improve comparability of data with the non-ligated (linear dimer) peptide. Results are means from triplicate experiments with standard deviations.

Membrane disruption was assayed by liposome leakage. The assay enables monitoring of membrane permeabilizing properties relative to introduced structural modifications with high specificity. *E. coli* and *Saccharomyces cerevisiae* membrane lipids were reconstituted into large unilamellar vesicles, trapping a self-quenching fluorescent marker. These liposomes carry a substantial negative charge because of the high proportion of anionic phospholipid head groups. In both these model membranes, anionic phospholipids account for approximately one third of total lipids, which for *E. coli* liposomes results in a zeta potential of −41 mV similar to the bacterial counterpart (Strömstedt et al., 2009b). KR-12 induced leakage from the *E. coli* liposomes, but with much reduced potency compared to LL-37 (Figure 3A). A 38-fold higher concentration of KR-12 than for LL-37 was required to reach 50% leakage (Table 2). Retro-KR12 had a significantly lower level of membrane leakage activity on the *E. coli* liposomes, rendering less than half the amount of leakage compared to KR-12, supporting the trend observed in the antibacterial assay.

**TABLE 2:** Comparison of antimicrobial activity (MIC), cytotoxicity (IC_50_), and hemolysis (HC_50_).

Peptide	MIC bact.^a^ (μ M)	MIC fung.^b^ (μ M)	EC50^c^ bact. (μM)	EC50^c^ fung. (μM)	HC50^d^ (μ M)	IC50^e^ (μ M)	HC50/MIC	HC50/IC50
LL-37	0.31	1.25	0.65	0.63	>80	10	>256	8
KR-12	2.5	10	≈25	0.95	>80	>80	32	>1
cd4	0.63	2.5	0.50	0.47	>40	5.1	64	7.8
cd4(Q5K,D9K)	0.63	1.25	0.30	n.d.	10	1.0	16	10

*^*a*^MIC against *E. coli*. ^*b*^MIC against *C. albicans*. ^*c*^Liposome leakage. ^*d*^Hemolysis against erythrocytes. ^*e*^Cytotoxicity against lymphoma cells.*

Both linear and cyclic dimers were about 50-fold more potent than monomeric peptides on bacterial liposomes, irrespectively of Cys to Ala conversions in the linker. The four-residue linker gave higher activity compared to three or two residue linkers, both for tandem and reversed tandem repeats, but the direction of sequences did not exhibit any clear trend regarding influence on activity (Figure 3B). No obvious advantage of cyclization could be observed as both cyclic and linear dimers with four residue linkers showed similar EC50:s of 0.45–0.5 μM. However, the membrane permeabilization of the cyclic dimers could be further enhanced by increasing the net charge, as shown by cd4(Q5K,D9K). This peptide has an EC50 of 0.3 μM, and stood out as the most active of the peptides tested in this study.

The weak and non-sigmoidal membrane activity of the KR-12 monomers on the generic bacterial composition (*E. coli* polar lipid extract) contrasted with that on the fungal counterpart (*S. cerevisiae* polar lipid extract with 30% ergosterol), where they exhibited strong activity with a sigmoidal concentration dependence (Figure 3C). However, the cyclic dimers and LL-37 behaved almost identical on fungal and bacterial liposomes.

### Dimerization Enhances Cytotoxic Activity

**FIGURE 4:**
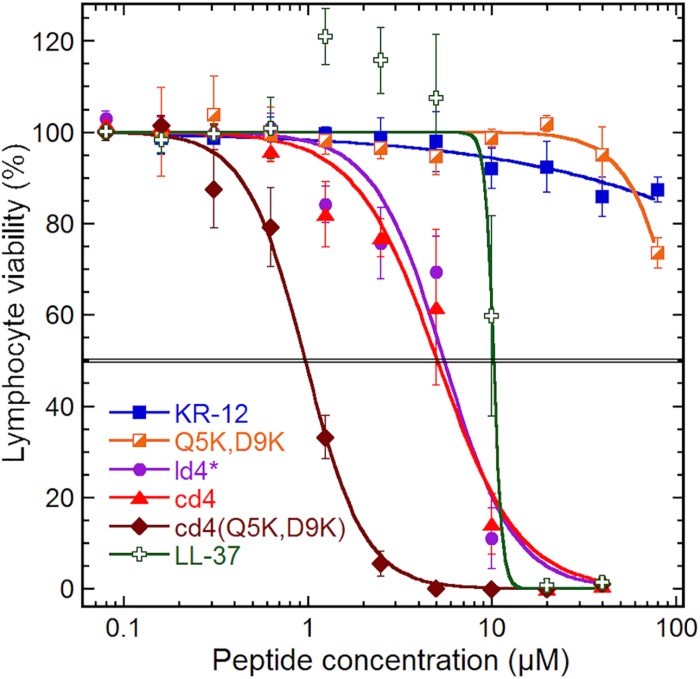
Cytotoxicity. Cytotoxicity on a human lymphoma cell line (U-937 GTB). The cell viability derives from the hydrolysis of the probe fluorescein diacetate by esterases in cells with intact plasma membranes. Results are means from triplicates with standard deviation, and the IC50 level is highlighted by a double line. The dose–response curve for LL-37 has been constrained within 100% to compensate for elevated fluorescein hydrolysis by intermediate LL-37 concentrations. The data for KR-12, Q5K,D9K and LL-37 are from our previous work (Gunasekera et al., 2018), but tested in parallel with the dimers and included here for consistency.

The monomer KR-12 lacked cytotoxicity against the human lymphoma cell-line with only 13% loss of cell viability observed at the maximum concentration of 80 μM tested (Figure 4), and monomer mutants showed similar or lower activity. In comparison, LL-37 was cytotoxic to the lymphoma cells with an IC50 of 10 μM. Dimerization resulted in significant lymphoma cell toxicity (1–5 μM) irrespective of whether the dimers were linear or cyclic. Notably, cd4(Q5K,D9K) showed the highest cytotoxicity (IC50 1 μM) of all the analogs tested on the cancer cell line. However, in the hemolysis assay, the dimers were 7 to 10-fold less toxic to healthy erythrocytes, possibly indicating that the membrane composition effects from prolonged and fast cell division of the cancer cells might make them more susceptible. Both KR-12 and LL-37 showed no hemolysis at the highest concentrations tested (>80 μM).

### Cyclic Dimers Have Enhanced Biological Stability

**FIGURE 5:**
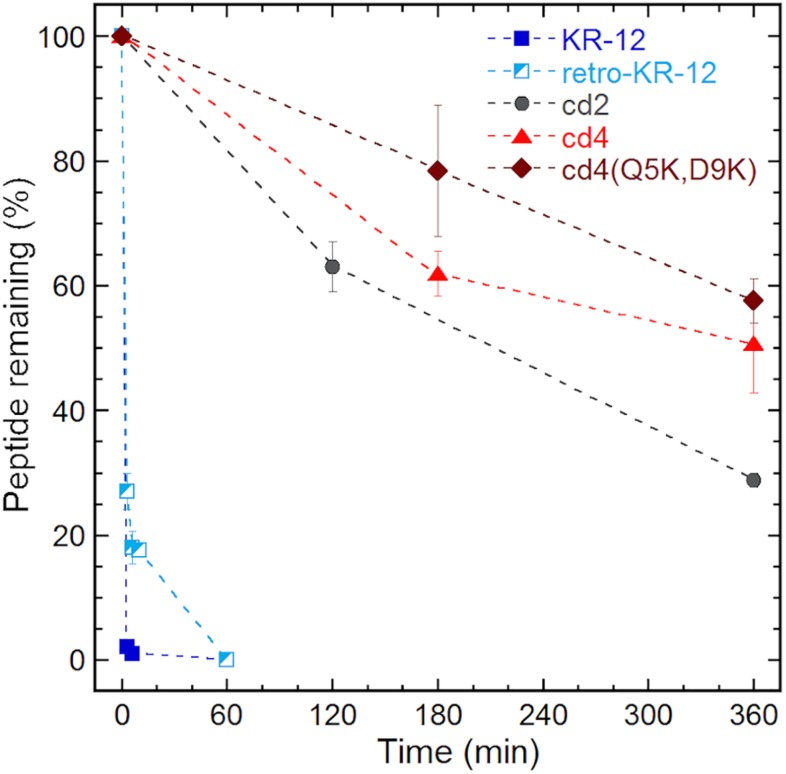
Serum stability of linear and cyclic peptides.

Proteolytic stability of the peptides were determined in diluted human serum (1:4 v/v, serum:PBS) at 37°C followed by LC-MS analysis. To examine the intact peptide after serum incubation, aliquots of each incubation mixture were taken at each time point for LC-MS analysis (Figure 5). In our previous studies we have shown that Ala and Lys analogs break down in 10 min (Gunasekera et al., 2018). In comparison to KR-12 and its derivatives, about 20% of retro-KR-12 peptide remained after 10 min, but it could not be detected after 20 min; R1, R11, and K13 were identified as cleavage sites by the analysis of degradation products in serum.

Even at time 0, LL-37 and ld4^∗^ peptides gave weak MS signals with high background noise, indicating that there was little free peptide, and peptides being rapidly degraded. Compared to KR-12, a significant proportion of free un-degraded cyclic peptides appear to be present in serum from the start of incubation. Notably, around 50% of the cyclic peptides, cd4 and cd4(Q5K,D9K) remained even after 6 h of incubation in serum. The stability of cd2 was lower than cd4 and cd4(Q5K,D9K), but still around 30% of cd2 was observed after 6 h of incubation in serum. The cyclotide kalata B1, which was used as a control representing a natural family of ultra-stable cyclic peptides, retained 80% of its initial concentration after 24 h.

### Cyclic Dimers Show High Helical Content in Lyso-Phosphatidylglycerol Membrane Mimicking Environment

**FIGURE 6:**
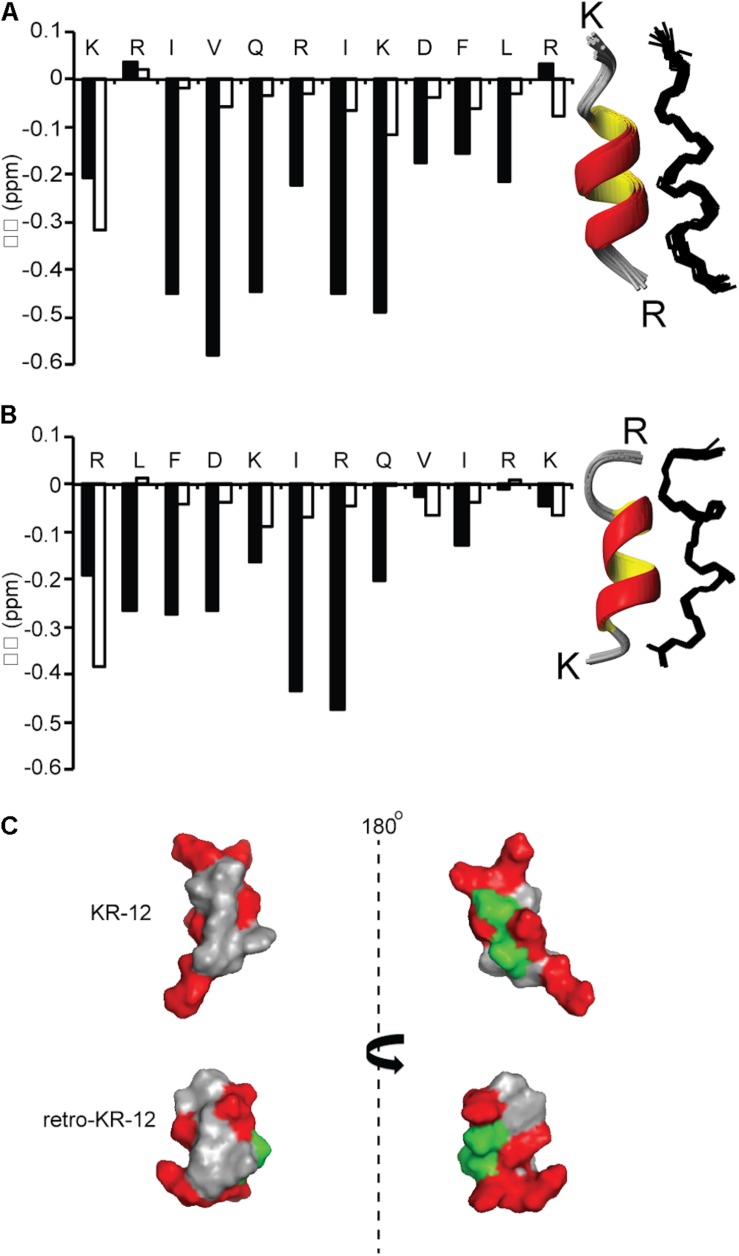
Comparison of secondary and three dimensional structures of KR-12 and retro KR-12. Secondary chemical shift (Δδ) was calculated by subtracting random coil αH chemical shift from the experimental αH chemical shift. Stretches of negative values (<0.1) represent alpha-helical structure. **(A)** KR-12 **(B)** retro-KR12; white bars represent the peptide in H_2_O:D_2_O (90:10 v/v) and black bars represents the peptide H_2_O:D_2_O (90:10 v/v) together with deuterized SDS (1:40, peptide:SDS). The corresponding three dimensional structures are represented by superimposition of the 15 lowest-energy structures (Ribbon and Line structures). **(C)** Ribbon structure with the lowest energy and surface representation of the structure; hydrophobic residues are highlighted in gray, polar residues in green and positively charged in red. The left view has similar orientation as the ribbon model and the right view is from the back. In the current paper, the KR-12 structure was calculated with higher number of distant constrains, i.e., 245 distant constrains (81 sequential restrains, 54 medium range restrains, 16 H bond restrains) in comparison to 86 distant restrains (37 sequential, 32 medium range, no H bonds) used in a previous study. The RMS deviation from mean coordinate structure for backbone atoms is similar for KR-12 in SDS (0.2 ± 0.05) and KR-12 in D8PG (0.24). Stereochemical quality of the KR-12 structure in SDS is 94 ± 6.11% residues in the most favored Ramachandran region in contrary to 90% residues reported for KR-12 in D8PG (Wang, 2008).

NMR spectroscopy was used to determine structures of KR-12 monomers and cyclic dimers. The spectra of KR-12 and retro-KR-12 in 90% H_2_O/10% D_2_O showed limited dispersion of peaks, indicative of unstructured conformations in aqueous environment. In contrast, spectra improved for KR-12 and retro-KR-12 when SDS-micelles were added to the samples, allowing resonance assignments using sequential assignment strategies and two dimensional TOCSY and NOESY experiments. Three dimensional solution structures were subsequently calculated with CYANA 3.0 using inter-proton distances derived from NOEs and dihedral angles derived from a TALOS-N chemical shift analysis. These calculations show that structurally well-defined alpha-helical conformations are present for both peptides in membrane simulated SDS environment. The structural ensembles for both peptides (Figure 6) have high stereochemical quality, with low numbers of steric clashes and no Ramachandran outliers. The structural statistics are given in Table 3 and the atomic coordinates for KR-12 and retro-KR-12 have been deposited in the Protein Data Bank (PDB) with accession number 2na3 and 2nal, respectively.

**TABLE 3:** Structural statistics for the family of the 20 lowest energy structures with highest MolProbity scores for KR12 and retro-KR12.

Parameters^1^	KR12	Retro-KR12
**Distance restraints**		
Intraresidue, | i-j = 0	94	98
Sequential, | i-j| = 1	81	102
Medium range, 1 < | i-j| < 5	54	107
Long range, | i-j| = 5	0	4
Hydrogen bond restraints^2^	16	16
Total	245	327
**Dihedral angle restraints**		
Φ	8	6
Ψ	8	6
Total	16	12
**Violations from experimental restraints**		
Distance constraints (>0.1 Å)	0	0
Dihedral angle constraints (> 5°)	0	0
**Stereochemical quality**		
Residues in most favored Ramachandran region, %	95.3 ± 7.2	94 ± 6.1
Ramachandran outliers, %	0	0
Unfavorable side chain rotamers, %	27.2 ± 5.7	23.3 ± 9.7
Clashscore, all atoms^3^	9.9 ± 2.5	5.83 ± 3.7
Overall Molprobity score^4^	2.79 ± 0.28	2.51 ± 0.55
**RMS deviations from mean coordinate structure (Å)^5^**		
Backbone atoms	0.31 ± 0.12	0.2 ± 0.05
All heavy atoms	1.12 ± 0.18	0.92 ± 0.11

*^1^Only structurally relevant restrains, as defined by CYANA are included. ^2^For 8 H-bonds (two restraints were used per hydrogen bond). ^3^Defined as the number of steric overlaps >0.4Å per thousand atoms. ^4^According to Molprobity (http://molprobity.biochem.duke.edu) ^5^atomic r.m.s.d values are for residues 1–12.*

NMR data were also recorded for the cyclic dimers cd4, cd4(Q5K,D9K), and 2retro-cd4, however spectra showed limited dispersion and broad lines for all peptides, indicative of disordered conformations. Unlike for KR-12 and retro-KR-12, addition of SDS and lyso-phosphatidylglycerol micelles did not change NMR spectra for the cyclic peptides, thus we turned to circular dichroism (CD) measurements to obtain complementary evidence for peptide conformations.

**FIGURE 7:**
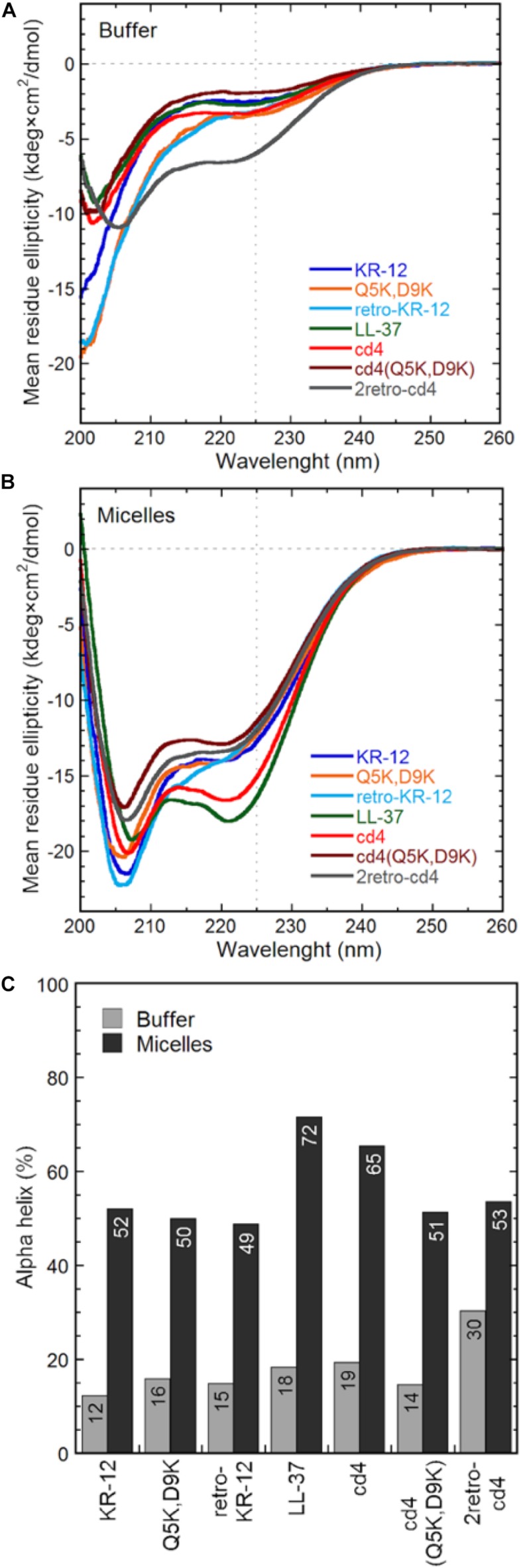
Peptide general structure from circular dichroism. The mean residue molar ellipticity curves from KR-12 analogs and cyclic dimers are shown from **(A)** peptides in Tris buffer only and **(B)** peptides in 16:0 lyso-phosphatidylglycerol (lyso-PG) micelles at a 1:1 peptide-to-micelle ratio (eq. to a 1:200 peptide-to-phospholipid ratio). Micelles of this type have physiologically relevant anionic headgroups and a micelle diameter similar to bacterial membrane core thickness. The amount of alpha helix **(C)** is determined from the signal intensity at 225 nm using a poly-Lys reference.

CD spectroscopy measurements were done in 10 mM Tris-buffer at pH 7.4, or with 16:0 lyso-phosphatidylglycerol (lyso-PG) micelles. These micelles are meant to representing the negatively charged microbial membrane environment, having the same anionic lipid headgroup and hydrophobic core thickness as *E. coli* membranes. All peptides adopted predominately random coil confirmation in Tris-buffer (Figure 7A). In contrast, the CD spectra show clear alpha-helical structure in presence of lyso-PG, where the dimers had 49–65% alpha-helical content (Figure 7B). The cd4 peptide was the dimer with highest alpha-helical content (65% helical content), and almost as alpha-helical as LL-37 itself, despite the turns necessitated by cyclization. Notably, introducing four positive charges to cd4 as in cd4(Q5K,D9K) resulted in a drop of helical content from 65 to 51%. The α-helical content (%) of each peptide in Tris-buffer and micelles is shown in Figure 7C.

## Discussion

In the current work, a series of head-to-tail cyclic peptide dimers, based on the twelve-residue-long KR-12 derived from human cathelicidin LL-37, were investigated for effects on antimicrobial activity and peptide stability. Dimerization significantly enhanced the antimicrobial activity, most notably against *P. aeruginosa* and *S. aureus*, with 8 to 16-fold enhancement in activity compared to KR-12. Although cyclization *per se* did not confer substantial increase antimicrobial activity compared to linear dimers, it significantly improved serum stability of the peptides. A change in the direction of the sequence from KR-12 to retro-KR-12, resulted in marginal decrease in antibacterial activity, but increase in antifungal activity. However, both antibacterial and antifungal activities increased for the dimers containing either one or two retro-KR-12 units. The cytotoxicity against lymphoma cells (IC50: 1 μM) observed for cd4(Q5K,D9K), a cyclic dimer rich in cationic charge content, indicated the importance of net positive charge in the interaction with cancer cell membranes. Together with 50% hemolysis for this analog occurring at 10-fold higher concentration (10 μM), this can serve as a reminder of the possibility of AMPs potential for anti-cancer applications.

### Cyclic Dimers Display Potent Activity, Increased Stability, and a Broad Therapeutic Window

KR-12 was selected as template for peptide design because of its size, structure and potent antimicrobial activity (MIC of KR-12 range from 2.5 to 10 μM against the current test organisms) and its low hemolytic activity (HC50 > 80 μM). For comparison between peptides in the present work, the ratio between MIC and HC50 is viewed at as an estimate of its “therapeutic window.” Dimerization substantially enhances antibacterial activity with factors of 8–16 against both *P. aeruginosa* and *S. aureus*, reaching a level comparable to the activity of the parent peptide LL-37. The results also demonstrate that dimers have a larger therapeutic window, for example HC50 was observed at a 64-fold higher concentration than the corresponding MIC against *E. coli* for one of the most promising cyclic dimers, cd4. The increased activity may be explained by the fact that dimerization can be viewed as not only a 2-fold increase of the number of active peptide in the system, but also to artificially increase the local peptide concentration on the membrane. For an AMP to disrupt the cytoplasmic membrane, the AMP molecules must first accumulate on the membrane surface to reach a concentration threshold where the membrane integrity is compromised (Oren et al., 1999; Xhindoli et al., 2014). After the initial interaction with the membrane, AMPs typically undergo self-association, multimerization and peptide-lipid associations to form structures like barrel-stave and toroidal pores, or other less defined structures that facilitate membrane permeabilization (Shai, 1999). Hence, it is well known that local concentration have a direct effect on activity, but the effect in the current work shows that this can be artificially achieved by covalent dimerization given the right circumstances.

The cyclic dimers displayed greatly enhanced stability in serum with more than 50% of an amount remaining after 6 h of incubation. In comparison, it was not possible to detect LL-37 or linear dimer ld4^∗^ in serum even at time 0, demonstrating their rapid degradation. Thus, even if the antibacterial potency exhibited by the cyclic dimers is comparable to LL-37, their enhanced stability in serum give them superior properties as a potential antibiotic template, and likely make them advantageous in a therapeutic context such as in treatment of a bacterial infection *in vivo*. In addition, the broad-spectrum activity displayed by the cyclic dimers is applicable in certain therapeutic areas, such as complicated skin and soft tissue infections, where a rapidly increasing incidence of polymicrobial infections involving both Gram-positive and Gram-negative organisms is reported. Stability can likely be even further improved by the use of linkers that even better preserve the structures of monomers.

### Electrostatic Interactions and Helical Structure Are Functionally Important

KR-12 monomers adopt a well-defined alpha-helical structure in simplistic membrane-mimicking environment (SDS micelles) to display distinct hydrophilic and hydrophobic surfaces (Figure 6). The alpha-helical structure adopted in SDS in the current paper is very similar to the KR-12 structure adopted in D8PG micelles (2 mM, pH 5.4, 25°C) (Wang, 2008). A clear alpha-helical structure between Ile3 to Leu11, forming an amphipathic structure with polar/charged residues on one surface and hydrophobic residues on the other is shown in Figure 6. A similar conformation adopted in bacterial membranes presumably assists its membrane lytic property with resulting antibacterial activity. However, cyclic dimers did not display defined structures either in water or in SDS. Using NMR, the assignment of residues for the dimers was still difficult in lyso-PG environment presumably due to the disordered linker regions and overlapping signals. However, 49–65% alpha-helical content was observed during CD analysis for dimers in membrane mimicking lyso-PG environment. Thus, it appears that for the dimers, both charge interactions and helical structure play a role in influencing antimicrobial activity. All dimers have a hydropathy index around −0.5 and a net charge of +8, except cd4(Q5K,D9K) which has a net charge of +16. High density of similarly charged groups is detrimental to helix formation (Murai and Sugai, 1974). This is demonstrated by the drop in helical content from 65 to 51%, as judged by CD spectroscopy, in by the introduction of four Lys residues in cd4 to cd4(Q5K,D9K) (Figure 7C). The cyclic dimer cd4(Q5K,D9K) is also substantially more potent than the monomeric Q5K,D9K in terms of membrane disruption. The fact that this is the most active dimer in the antimicrobial, liposome leakage and cytotoxicity assays indicates the importance of cationic charge interaction with the negatively charged membranes.

### Dimers Disrupt Lipid Membranes With Enhanced Efficacy

All dimers displayed higher antimicrobial activity than KR-12 and they are also more potent in the liposome assay. This indicates that membrane permeabilization is the main mechanism responsible for bactericidal and fungicidal activity by the dimers. Membrane disrupting AMPs act in a concentration dependent manner, where permeabilization occurs when a certain local membrane concentration is reached, either by direct peptide multimerization or by cumulative stress on the bilayer coherency. In comparison to the 4 to 16-fold increase activity in the antibacterial assays, the dimers displayed nearly 50-fold enhancement in leakage in the corresponding liposome model. However, although membrane permeabilization appears to be the main mode of action, it should be pointed out that the liposome model does not represent the full complexity of a bacterial membrane, nor does it take into account other factors and mechanisms that may modulate antibacterial activity. Over the past few decades, a large number of studies have reported on the activation of a receptor and/or components of downstream signal induction pathway of LL-37, although the mechanistic details underlying receptor activation is poorly understood at present (Verjans et al., 2016). In light of these findings, it is possible both KR-12 and its variants, including cyclic dimers, have other targets besides membranes.

Comparing the various dimer constructs on bacterial liposomes, it appears that in terms of membrane permeabilization the cyclization process does not affect activity as shown by the overlapping leakage curves for ld4^∗^ and cd4^∗^ (Figure 3A). Similarly, the direction of monomer sequences did not affect membrane permeabilization in any discernable way (Figure 3B). The factor most relevant for optimized bacterial membrane permeabilization within the set of dimers was linker length, where the long four-residue linker proved more successful than the shorter ones in this perspective. However, the beneficial effect of longer linker was not very pronounced, and neither was this effect mirrored by any substantial advantage in the antibacterial assay.

The most potent dimer was cd4 (EC50: 0.5 μM). Upon utilizing the double-lysine substituted analogue in lieu of the native KR-12 sequence, the same type of dimer reached an EC50 of 0.3 μM, which can be compared with 0.7 μM for LL-37. The cd4 dimers and LL-37 exhibited almost identical activity on liposomes of fungal and bacterial lipid extracts. Noteworthy is however that the monomers display ideal sigmoidal dose response curves on the fungal membranes as compared to the incomplete permeabilization achieved on the bacterial counterpart. In this case the dimers are just 2–3 times as potent as the monomers. The 23-fold difference in EC50 for KR-12 in either liposome system is not mirrored in the antimicrobial assay (Table 2), and supports the previous proposition that membrane permeabilization is not the sole mechanism of action for the KR-12 monomer on bacteria (Gunasekera et al., 2018).

### Do Cyclic Dimers Show Promise for Development Into Stable Therapeutic Leads?

It is possible to envision potential applications for stable AMPs, such as topical treatment of wounds and ophthalmic uses. Furthermore, the high prevalence of infections caused by surgical devices such as catheters has created a demand for stable antimicrobial indwelling device coatings, but perhaps most importantly as novel tools to combat the crisis of resistance development. Thus, the cyclic dimers developed in the current work may afford several opportunities under a number of clinical settings due to their broad-spectrum antimicrobial activity and enhanced stability.

A minimalized scaffold will be more attractive in terms of reduced cost for synthesis and development. Notably, in cd2 dimer, the size was significantly reduced (28 residues) and so was the sequence variability (only 11 different amino acids) compared to LL-37 (37 residues in total and 16 residue types). The post-synthesis works up for head-to-tail cyclization cannot be avoided in the current synthesis scheme, but biological methods such as enzyme-mediated cyclization are currently emerging. Although, low stability is currently a drawback in the linear dimers, the ld4 peptide has antibacterial activity levels parallel to LL-37. Notably, its antifungal activity is superior to LL-37 and also better than the cyclized dimers.

AMPs may also find other applications. In fact, the increase in membrane disruption and cytotoxicity conferred by dimerization to a level surpassing LL-37 warrants further studies. In particular, cd4(Q5K,D9K) displayed the highest cytotoxicity (IC50: 1 μM). Tumor cells have higher anionic potential on the outer leaflet due to accumulation of phosphatidylserine (Utsugi et al., 1991; Riedl et al., 2011), which may make AMPs more cytotoxic toward tumor cells compared to healthy cells. This was in fact evident from the data as cd4(Q5K,D9K) showed a HC50/IC50 of 10, which indicates that only at 10-fold higher concentration than its IC50 it is toxic to normal erythrocytes. In comparison, cd4, which has a net positive charge of +8 displayed relatively low anti-cancer activity with a LC50 of 5.1 μM and consequently low hemolysis (>40 μM). Notably, the HC50/IC50 for cd4 is around 7 indicating that significant unspecific cell toxicity occurs only above a 7-fold higher concentration than LC50.

A strong correlation between antitumor activity and net positive charge has been demonstrated with at least +7 was required for a strong cytotoxic activity toward the tumor cells (Yang et al., 2004). In another study, CB1, a cecropin-derived peptide was shown to have high anti-cancer activity and low hemolysis, and the net positive charge of +12 was proven to be important for its activity (Wu et al., 2009). Consequently, highly membrane active AMPs could also find potential applications in cancer therapy and the cyclic dimer template developed in the current work may provide a promising template for further optimization of anti-cancer activity.

## Conclusion

The design of novel host defense peptides requires the optimization of multiple parameters. One of the important hurdles in antibacterial drug lead design is the concern of stability. Here, we showed that backbone cyclization of KR-12 dimers substantially improves biological stability of KR-12, and dimerization confers higher local concentration of the peptide to mediate enhanced antimicrobial activity. With better understanding of the molecular basis of membranes and more realistic recapitulations of host environments, these first-generation analogs provide the basis for development of further improved AMPs.

### Experimental Section

All Fmoc protected amino acids and Boc-Cys(Trt)-OH were purchased from PepChem (Tubingen, Germany) or Iris Biotech (Marktredwitz, Germany). The following Fmoc amino acids were used: Fmoc-Ala-OH, Fmoc-Arg(Pbf)-OH, Fmoc-Asp(OtBu)-OH, Boc-Cys(Trt)-OH, Fmoc-Gln(Trt)-OH, Fmoc-Gly-OH, Fmoc-Ile-OH, Fmoc-Leu-OH, Fmoc-Lys(Boc)-OH, Fmoc-Phe-OH, Fmoc-Val-OH. Following resins were used in synthesis: Tentagel R Ram Rink-type resin (0.18 mmol/g, Peptide International, Louisville, KY, United States), Fmoc-Arg(Pbf)-Novasyn TGT (0.2 mmol/g, Novabiochem, Stockholm, Sweden) and Fmoc-Lys(Boc)-Novasyn TGT (0.2 mmol/g, Novabiochem). Di- Fmoc-3,4-diaminobenzoic acid was purchased from BioNordika, Stockholm, Sweden or Iris Biotech. 2-(1H-Benzotriazole-1-yl)-1,1,3,3-tetramethyluronium hexafluorophosphate) HBTU) was purchased from PepChem. *N,N*-dimethylformamide (DMF) was from Thermo Fisher Scientific (Waltham, MA, United States) or Saveen & Werner (Limham, Sweden). HPLC grade acetonitrile (AcN) was from (West Chester, PA, United States). Diethyl ether was purchased from Merck (Darmstadt, Germany). Diisopropylethylamine (DIPEA), Guanidine hydrochloride (GnHCl), trimethylsilylisopropane (TIPS), piperidine, trifluoroacetic acid (TFA), trichloroacetic acid (TCA), Urea, Tris (2-carboxyethyl) phosphine hydrochloride (TECP.HCl), 4-mercaptophenylacetic acid, 4-Nitrophenyl chloroformate, Gluthathione reduced, VA-044 (2,2′-Azobis[2-(2-imidazolin-2-yl)propane]dihydrochloride), Phosphate Buffered Saline and Human serum from human male AB plasma (United States origin, sterile filtered) were purchased from Sigma-Aldrich, Stockholm, Sweden. Sephadex G-15 for size exclusion chromatography was from GE Healthcare Life Sciences, Uppsala, Sweden. Tryptic Soy Broth was from (Merck KGaA, Darmstadt, Germany).

### Bacterial and Fungal Strains

The three bacterial strains: *Escherichia coli* ATCC 25922, *Pseudomonas aeruginosa* ATCC 27853 *Staphylococcus aureus* ATCC 29213 as well as the fungal strain *Candida albicans* ATCC 90028 derived from clinical wound infections were obtained from the Department of Clinical Bacteriology at Lund University Hospital.

### Peptide Synthesis

Peptide synthesis was performed on a CEM Liberty 1 (CEM Corporation, Matthews, NC, United States) automated microwave-assisted peptide synthesizer using Fmoc/tBu chemistry with piperidine (20% v/v) as the Fmoc deprotecting agent. All peptides were synthesized on a 0.1 mmol scale using a 5-fold molar excess of Fmoc-protected amino acids, activated by a 5-fold excess of HBTU in the presence of DIPEA (10 equiv, 2 M). All the linear peptides were assembled using the already established protocol (Gunasekera et al., 2018). Cyclic peptides were synthesized on the Tentagel Rink amide resin. Coupling of the Dbz and first C terminal amino acid Gly in cyclic analogs were carried out manually and rest of the synthesis was continued on the automated microwave peptide synthesizer using conditions specified earlier (Gunasekera et al., 2013). Following synthesis, the resin was washed well with dry DCM and dried under N_2_.

Each dimeric peptide was synthesized by Fmoc-SPPS as a single chain of amino acids by arranging two KR-12 units one after the other or by linking one KR-12 unit with its retro KR-12 peptide; the synthesis was initiated at the C-terminal diaminobenzoic acid (Dbz) group and an N terminal Boc-Cys was incorporated.

### Conversion of Dbz Peptides to Nbz Peptides

To obtain the peptides intended for cyclization, the resin bound peptide containing the Dbz linker was acylated using 4-Nitrophenylcholoroformate in DCM (16 equiv, 0.05 M, 25°C, 60 min). The resin was washed with DCM, followed by DMF and activated with 0.5 M DIPEA in DMF (195 equiv., 0.5 M, 20 min).

Following synthesis (directly after synthesis for linear peptides and after Nbz conversion for peptides intended to be cyclized), a sample of the resin was taken out and cleaved with a mixture containing TFA/TIPS/water (95:2.5:2.5, 1.5 h). The cleaved peptide was filtered from resin and dried down to 0.5 ml under N_2_, precipitated by addition of cold diethyl ether and centrifuged at 1700 rpm for 10 min. The precipitate was collected by centrifugation, re-dissolved in 50% AcN/0.05% TFA and freeze dried.

### Cyclization/Native Chemical Ligation

KR-12 cyclic dimers were obtained using a Native Chemical Ligation protocol (NCL) (Gunasekera et al., 2013). A stock buffer (100 ml) of 0.159 M Na_2_HPO_4_.2H_2_O (Sodium phosphate dibasic) containing 6M GnHCl (Guanidine hydrochloride) was prepared. The cyclization buffer (25 ml) was prepared from the filtered stock buffer by adding 50 mM MPAA (4-mercaptophenlacetic acid), 20 mM TCEP.2HCl (tris-2-carboxyethylphosphine hydrochloride) and adjusting the pH to 7.0 – 7.1 with 2 M NaOH. The cyclization buffer was degassed and the Nbz peptides (0.3–0.6 mM) were incubated in the cyclization buffer (pH 7–7.1) for 24 h at 25°C. After 24 h the cyclization buffer was eluted through a packed size exclusion column (Sephadex G-15, GE Healthcare Lifesciences) using 30% AcN in water. The fractions matching the expected masses for the cyclic peptides were identified by MS and freeze dried.

### Desulfurization

A standard desulfurization protocol was used (Wan and Danishefsky, 2007). A stock buffer (20 ml) containing 0.2 M Sodium phosphate (prepared from 0.712 g Na_2_HPO_4_.2H_2_O and 0.552 g of NaH_2_PO_4_.H_2_O) and 6 M Guanidine HCl was prepared (pH 6.5). The following solutions (1 ml each) was prepared in stock buffer separately; 0.5M TCEP.2HCl, 200 mM VA-044, and 40 mM glutathione (reduced). The desulfurization buffer was prepared by mixing 100 μl of VA-044, 500 μl of TCEP and 125 μl of glutathione (pH 6.5). The peptide dissolved in 500 μl of water was added to the desulfurization mixture (1-2 mM) and incubated for 3 h at 65°C. Following incubation, the desulfurization mixture was passed through a Sephadex PD- 10 column (GE Healthcare) equilibrated with 30% AcN in water. The fractions matching the expected mass for the desulfurized peptides were identified by MS and subsequently freeze dried.

### HPLC and MS

RP-HPLC was performed on an Akta Basic (GE Healthcare) and Shimadzu LC10 AD (Shimadzu, Japan) with detection at 215, 254, and 280 nm. Preparative HPLC was done on a 250 × 10 (i.d.) mm Phenomenex (Torrance, CA, United States) C18 column (5 μm) at a flow rate of 4 ml/min, and analytical HPLC were done on a 100 × 4.60 (i.d.) mm Phenomenex C18 column (2.6 μm) at a flow rate of 0.5 ml/min. Solvents A (10% AcN, 0.05% TFA in water) and B (60% AcN, 0.05% TFA in water) were used in a linear gradient from 0 to 100% solvent B over 70 min for the preparative HPLC. Solvents A (0.05% TFA in water) and B (0.05% TFA in AcN) was used in a linear gradient from 5 to 90% B over 35 min was used for analytical HPLC. LC–MS was performed on a nano Acquity Ultra Performance LC coupled to a micromass Q-TOF detector. A flow rate of 0.3 μl/min was used on a Nano Acquity UPLC BEH C18 column (1.7 μm, 250 × 0.075 mm) using solvents A (0.1% FA in water) and B (0.1% FA in AcN). A linear gradient from 0 to 90% B in solvent A was used for analysis over 75 min.

### Antibacterial Assay

Antimicrobial activities of the peptides were evaluated using the two-step microdilution assay (Strömstedt et al., 2017), which is designed for testing AMPs without activity-inhibiting components. Briefly, the microbial suspension was washed twice with 10 mM Tris buffer (set to pH 7.4 at 37°C). 96-well plates (U-shaped, untreated polystyrene) were prepared with peptide 2-fold serial dilutions in Tris buffer. The culture density was quantified by OD_600_, diluted in Tris buffer and 50,000 cells aliquoted to each well in the 96-well plate to a final 100 μl volume. After 5 h of aerobic incubation at 37°C, 5 μl of 20% TSB medium was added to each well (resulting in 1% TSB) and the plates were re-incubated. Growth-inhibition was monitored when controls produced pellet sizes of 2 mm in diameter (close to growth saturation), which equates to 5, 8, 10, and 12 h for *E. coli, S. aureus, P. aeruginosa, and C. albicans*, respectively. The MIC values were defined as the lowest peptide concentration that fully inhibited visible bacterial growth. Cells from MIC-wells did not exhibit growth after longer incubation times or through resuscitation attempts in fresh media. The MIC-values in Table 1, are medians from triplicate experiments, while the data presented in the Figure 2 are the same values relative to that of KR-12. Deviations between experiments did not exceed one dilution factor. Please note that although this assay protocol gives less variation, it also gives lower MICs for AMPs compared to the standard broth microdilution assay in which the rich growth media conditions specifically inhibit much of AMP activity. The benchmark AMP LL-37 can thus be used here as a reference point for activity.

### Liposome Leakage Assay

The liposome production and the liposome leakage assay were performed similar to what has previously been described (Strömstedt et al., 2016). Polar lipid extract from *E. coli*, and *S. cerevisiae* (Avanti Polar Lipids, Alabaster, AL-US) were used, the latter supplemented with ergosterol (Sigma-Aldrich) at a 7:3 molar ratio. In brief, dry lipid films were deposited on round-bottom flask walls and re-suspended in Tris buffer containing of 100 mM 5(6)-carboxyfluorescein. Multilamellar structures and polydispersity were reduced by repeated extrusion through 100-nm polycarbonate membranes. Un-trapped carboxyfluorescein was removed by gel separation. Membrane permeability was measured by monitoring carboxyfluorescein efflux from the liposomes to the external low concentration environment, resulting in loss of self-quenching and an increased fluorescence signal. The leakage experiments were performed on a 96-well plate format. Wells were prepared with a 2-fold serial dilution of the peptides in Tris buffer, as well as controls without peptides (background) and the detergent Triton X-100 (maximum leakage). The plates were pre-heated to incubation temperature (37°C) and liposomes subsequently administered (to a final lipid concentration of 10 μM in 200 μl) by an automated dispenser. The effect of each peptide concentration on liposome integrity was monitored for 45 min, at which point the leakage had largely subsided. Results shown represent the mean from triplicate experiments with standard deviations, and are presented as percent of total leakage generated with Triton X-100 and subtraction of the baseline value. EC50-values are calculated from sigmoidal dose-response curves of leakage percentage as a function of the peptide concentration (log10).

### Cytotoxicity Assay

The cytotoxicity of the peptides was tested on the human lymphoma cell line U-937 GTB in a fluorometric microculture cytotoxicity assay (FMCA), described in detail previously (Sundström and Nilsson, 1976; Lindhagen et al., 2008). The cell line was maintained in RPMI 1640 complete medium (Sigma-Aldrich, St. Louis, MO, United States) supplemented with 10% heat inactivated fetal bovine serum, 2 mM glutamine, 50 (μg/ml streptomycin and 60 (μg/ml penicillin (all from Hy Clone, Cramlington, GB) and kept under standard incubating conditions (humidified atmosphere of 37(°C, 5% CO2) and continuously grown at log phase. Peptide 2-fold serial dilutions were prepared in 96-well plates to which 20, 000 cells in fresh growth medium were added (to a total volume of 200 (μl) and incubated for 72 hours. Control wells without peptide or with triton X-100 (representing complete cell death) was used to quantify the viability interval. The cells were washed with PBS, and fluorescein diacetate added to each well. After incubation for 40 minutes the fluorescence was measured at 485 nm excitation and 538 nm emission using a Fluoroscan II (Labsystems Diagnostics Oy, Helsinki, FinlandI). The IC50-values were calculated using sigmoidal dose response curves with nonlinear non-linear regression and 0/100 constraints in GraphPad Prism (GraphPad Software, San Diego, CA, United States). Each marker Figure 4 represents the mean viability as a percent of the control growth, with standard deviations from triplicate experiments.

### Serum Stability Assay

Peptide stability was assayed in diluted human serum. 25% human serum was centrifuged at 13,000 rpm for 10 min to remove lipids and the supernatant was collected and incubated at 37°C for 15 min. Here 8 μl of selected aqueous peptide stock solutions (200–250 μM) were incubated in 25% human serum (80 μl) at 37°C for the desired time points (0, 3, 6, 10 min for linear peptides and 0, 2 4, 6, and 24 h for cyclic peptides). Eighty μl of 6 M urea was added and incubated at 4°C for a further 10 min. 80 μl of 20% TCA was added and incubated at 4°C for a further 10 min. The samples were centrifuged at 13000 *g* for 10 min and the supernatant was analyzed by LC-MS.

### Hemolysis Assay

Peptides were dissolved in water and serially diluted in PBS to give 20 μl test solutions in a 96-well U-bottomed microtiter plate (Nunc). Human type A RBCs (red blood cells) were washed with PBS and centrifuged at 1500 *g* for 60 s in a microcentrifuge several times until a clear supernatant was obtained. A 0.25% suspension of washed RBCs in PBS (100 μl) was added to the peptide solutions. The plate was incubated at 37°C for 1 h and centrifuged at 150 *g* for 5 min. Aliquots of 100 μl were transferred to a 96-well flat-bottomed microtiter plate (Falcon) and the absorbance was measured at 405 nm with an automatic Multiskan Ascent plate reader (Labsystems). The amount of hemolysis was calculated as the percentage of maximum lysis (1% Triton X-100 control) after adjusting for minimum lysis (PBS control). Synthetic melittin (Sigma) was used for comparison. The hemolytic dose necessary to lyse 50% of the RBCs (HD_50_) was calculated using the regression constant from the linear portion of the hemolytic titration curve (GraphPad Prism software).

### NMR Spectroscopy

Freeze dried peptides (0.3–1 mM) were dissolved in 600 μl H_2_O/D_2_O (9:1, v/v) at pH 4.5 and one and two-dimensional spectra (^1^H TOCSY and ^1^H NOESY) were recorded at 298 K. A further set of two dimensional spectra (^1^H TOCSY, ^1^H NOESY and ^15^N HSQC) were obtained after adding deuterized SDS (peptide:SDS, 1:40) and lyso-phosphatidylglycerol micelles to the samples. A series of ^1^H 1D/TOCSY spectra monitoring hydrogen-deuterium exchange as well as a ^1^H-^13^C HSQC spectrum was obtained at 298 K after dissolving the freeze-dried peptide in 100% D_2_O. All data, including TOCSY (mixing time 80 ms), NOESY (mixing time 200 ms), ^13^C-HSQC and ^15^N-HSQC were recorded and processed using Topspin (Bruker). Generally, 4,096 data points were collected in the F2 dimension and 256 (128 complex) points in F1, with 512 increments of 8 scans over 11,194 Hz.

### Structure Calculations

Resonance assignments of KR-12 and retro-KR12 were obtained using sequential assignment strategies. Once the chemical shift assignments were identified from TOCSY spectra, inter-proton distances were derived from all identified NOE cross peaks by peak-picking and manual integration in XEASY (Bartels et al., 1995). Then an ensemble of structures was calculated automatically using CYANA 3.0 (Güntert et al., 1997); the tolerances used for assigning NOESY cross peaks were 0.020 and 0.020 ppm in the F1 and F2 dimensions respectively. TOCSY, NOESY, DQF-COSY, and HSQC spectra were analyzed using the program package CARA (Keller, 2004). ^1^Hα, ^13^Cα, ^13^Cβ and ^15^N chemical shifts were used to predict peptide backbone φ/ψ torsion angle constraints from TALOS-N chemical shift analysis (Shen and Bax, 2013). Hydrogen bond restraints were identified by long-range NOE correlations and D_2_O exchange experiments in conjunction with preliminary structures. Thus, hydrogen bond restraints of 1.8–2.0 Å and 2.7–3.0 Å were used for the H_N_-O and N-O distances, respectively, in subsequent rounds of structure calculations. The three-dimensional structures were then generated using MOLMOL (Koradi et al., 1996), surface structures were produced using PYMOL the PyMOL (Molecular Graphics System, Version 2.0 Schrödinger, LLC) and structure qualities were validated using MolProbity (Chen et al., 2010).

### Circular Dichroism

The alpha-helical contribution to the secondary structure of the peptides was determined using a JASCO J810 spectropolarimeter (JASCO Corporation, Easton, MD, United States) monitoring changes in the 200–260 nm range in 10 mM Tris buffer (pH 7.4), with stirring in a 1 cm quartz cuvette. Signals from the peptides, at a concentration of 10 μM, were measured in buffer alone or with 10 mM 16:0 lyso-phosphatidylglycerol (lyso-PG) micelles. This resulted in a 1:1 peptide-to-micelle ratio (eq. to a 1:200 peptide-to-phospholipid ratio). Micelles of this type (in contrast to SDS micelles) have a physiologically relevant anionic headgroup and micelle diameter similar to bacterial membrane core thickness. Each spectrum depicted in Figure 7 is the mean from 10 accumulations at a rate of 50 nm/min. The solvent background contribution was subtracted for each wavelength as well as the baseline drift for each measurement (normalized at 260 nm, where no peptide signal is present). The quantification of alpha helix composition was calculated at 225 nm and compared to a poly-L-Lys reference (30–70 kDa from Sigma-Aldrich, St. Louis, MO, United States) in 0.1 M NaOH (100% alpha helix) and 0.1 M HCl (100% coil).

## Data Availability Statement

The datasets generated for this study can be found in the Protein Data Bank. Accession numbers for KR-12 and retro-KR-12 are 2na3 and 2nal, respectively.

## Author Contributions

All authors contributed to the design of the study, analysis of the data, and writing of the manuscript. SG and TM carried out the peptide synthesis. SG conducted the NMR experiments. TM and AS did the circular dichroism experiments. TM performed the antibacterial and antifungal assays. AS performed the cytotoxicity and liposome experiments.

## Conflict of Interest

The authors declare that the research was conducted in the absence of any commercial or financial relationships that could be construed as a potential conflict of interest.

## Footnotes

1 https://clinicaltrials.gov

## Abbreviations

AMP: antimicrobial peptide
CD: circular dichroism
Dbz: Di-Fmoc di-aminobenzoic acid
FMCA: fluorometric microculture cytotoxicity assay
FMOC: fluorenylmethyloxycarbonyl
BOC: tert-buyloxycarbonyl
HPLC: high performance liquid chromatography
MIC: minimum inhibitory concentration
MS: mass spectrometry
Nbz: *N*-benzymidazolinone
NMR: nuclear magnetic resonance
NOESY: nuclear Overhauser effect
RP: reverse phase
TOCSY: total correlated spectroscopy
TSB: Tryptic Soy Broth.
