# Supplementary material for: Backbone Cyclization and Dimerization of LL-37-Derived Peptides Enhance Antimicrobial Activity and Proteolytic Stability
Source: Front Microbiol. 2020 Feb 21;11:168. doi: 10.3389/fmicb.2020.00168 (PMC7046553; doi:10.3389/fmicb.2020.00168)
Supplement: Supplementary file 1 [file Data_Sheet_1.docx]

**Supporting Information**

**Backbone cyclization and dimerization of LL-37-derived peptides enhance antimicrobial activity and proteolytic stability**

*Sunithi Gunasekera^1#^, Taj Muhammad^1#^, Adam A. Strömstedt^1#^, K. Johan Rosengren^2^, Ulf Göransson^1#^*

^1^Pharmacognosy, Department of Medicinal Chemistry, Uppsala University, Biomedical Centre, Box 574, SE-75123 Uppsala, Sweden

^2­­­^The University of Queensland, School of Biomedical Sciences, Brisbane, QLD 4072, Australia

*^#^* Contributed equally

^*^Corresponding author: E-mail: [ulf.goransson@ilk.uu.se](mailto:ulf.goransson@ilk.uu.se), Department of Medicinal Chemistry, Biomedical Centre, Uppsala University, Box 574, Se-751 23 Uppsala, Sweden

| Item | Page number |
| --- | --- |
| Supplementary Table 1: Net charges, hydropathicity, masses and yield of the cyclic dimers | S2 |
| Supplementary Table 2. Net charges, hydropathicity and masses of the linear dimers . | S3 |
| Supplementary Figure 1: RP-HPLC and MS analyses of cyclic KR-12 analogues. | S4 |

**Supplementary Table 1. Net Charge, hydropathy, peptide masses and yields**

|  | **cd2** | **cd3** | **cd4** | **retro-cd2** | **retro-cd3** | | **retro-cd4** | **cd4**  **(Q5K,D9K)** | **2retro-cd4** | **cd4**  **(Q5K,D9K)** | **(Q5K,D9K)** | **(Q5K,D9K)** |
| --- | --- | --- | --- | --- | --- | --- | --- | --- | --- | --- | --- | --- |
| Net charge  Hydropathy  Dbz mass (calc.)* | +8  -0.507  3548.29 | +8  -0.5  3662.39 | +8  -0.491  3776.50 | +8  -0.507  3548.29 | +8  -0.5  3662.39 | +8  -0.491  3776.50 | | +14  -0.544  3803.067 | +8  -0.471  3776.50 | +14  -0.544  3803.067 | +14  -0.544  3803.067 | +14  -0.544  3803.067 |
| Dbz mass (exp.)* | 3548.46 | 3660.61 | 3773.89 | 3548.53 | 3660.31 | 3773.46 | | 3800.56 | 3773.46 | 3800.56 | 3800.56 | 3800.56 |
| Dbz peptide yield (%) | 16.27 | 65.47 | 57.45 | 24.01 | 29.54 | 32.44 | | 66.58 | 47.67 | 66.58 | 66.58 | 66.58 |
| Nbz mass (calc.)* | 3574.59 | 3688.39 | 3802.54 | 3574.59 | 3688.39 | 3802.54 | | 3829.07 | 3802.54 | 3829.07 | 3829.07 | 3829.07 |
| Nbz mass (exp.)* | 3573.52 | 3690.38 | 3801.03 | 3572.62 | 3689.17 | 3804.37 | | 3826.81 | 3803.66 | 3826.81 | 3826.81 | 3826.81 |
| Cyclic mass (calc.)* | 3396.17 | 3510.28 | 3624.38 | 3396.17 | 3510.28 | 3624.38 | | 3650.64 | 3624.38 | 3650.64 | 3650.64 | 3650.64 |
| Cyclic mass (exp.)* | 3398.47 | 3510.91 | 3624.82 | 3397.50 | 3508.42 | 3623.60 | | 3651.07 | 3624.05 | 3651.07 | 3651.07 | 3651.07 |
| Cyclic mass (calc.) | 3364.11 | 3478.22 | 3592.32 | 3364.11 | 3478.22 | 3592.32 | | 3618.58 | NP | 3618.58 | 3618.58 | 3618.58 |
| Cyclic mass (exp.) | 3364.96 | 3478.02 | 3592.03 | 3364.03 | 3478.93 | 3592.15 | | 3618.52 | NP | 3618.52 | 3618.52 | 3618.52 |

Calculated and experimental peptide masses highlighted with a * are prior to desulfurization. Hydropathy was calculated after desulfurization except 2retro-cd4.

The experimental [M+1]^1+^ are from the deconvoluted masses of corresponding [M+3]^3+^ ions. (NP, not performed)

**Supplementary Table 2. Net Charge, hydropathy and peptide masses**

|  | **Id4** | **retro-Id4** |
| --- | --- | --- |
| Net charge  Hydropathy  Linear mass (calc.)* | +8  -0.471  3641.54 | +8  -0.471  3641.54 |
| Linear mass (exp.)* | 3641.90 | 3642.01 |

The experimental [M+1]^1+^ are from the deconvoluted masses of corresponding [M+3]^3+^ ions.

**Supplementary Figure 1**

**
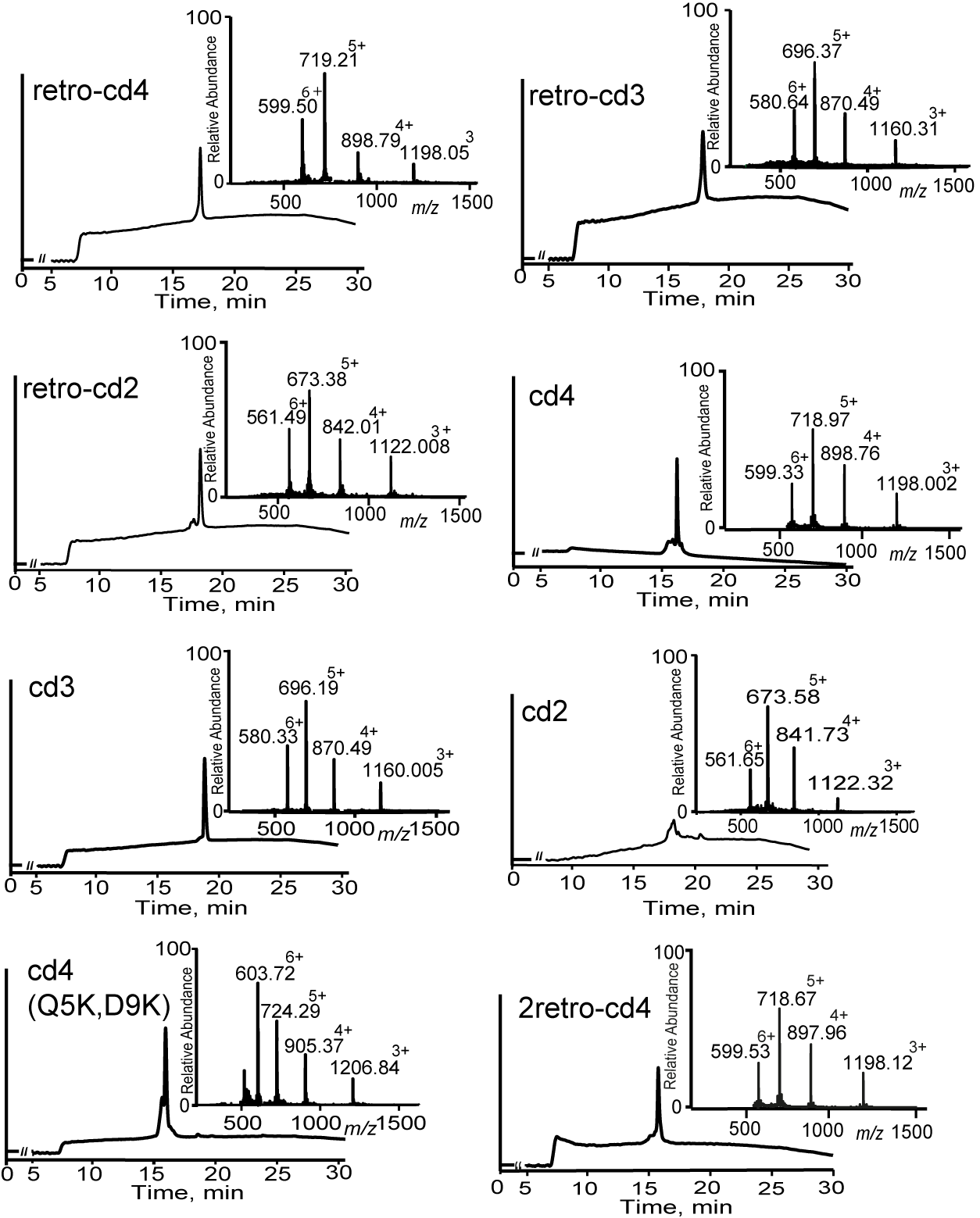
**

**Supplementary Figure 1: RP-HPLC absorbance at 215 nm and MS analyses of cyclic KR-12 dimers.**
